# Effect of Overfilled Solvent and Storage Time of Subcritical Extraction of *Jasminum sambac* on Yield, Antioxidant Activity, Antimicrobial Activity and Tentative Volatile Compounds

**DOI:** 10.3390/plants12030585

**Published:** 2023-01-29

**Authors:** Pattarin Supanivatin, Suwit Siriwattanayotin, Aluck Thipayarat, Paweena Ekkaphan, Jakrapop Wongwiwat

**Affiliations:** 1 Department of Food Engineering, King Mongkut’s University of Technology Thonburi, Bangkok 10140, Thailand; 2 Scientific and Technological Research Equipment Centre, Chulalongkorn University, Bangkok 10330, Thailand; 3 Department of Mechanical Engineering, King Mongkut’s University of Technology Thonburi, Bangkok 10140, Thailand

**Keywords:** phytonic extraction, HFC-134a, jasmine, essential oil, antioxidant, antimicrobial, volatile compounds

## Abstract

Essential oil from *Jasminum sambac* flowers has demonstrated the potential of antioxidant and antimicrobial properties. However, jasmine flowers contain only a small amount of essential oil; therefore, subcritical fluid extraction (SFE) with HFC-134a, one of the effective extraction methods for flower extraction, was performed in this study. The percentage of overfilled solvent and storage time of the flowers were varied during the extraction. Antioxidant potential, antimicrobial potential and tentative volatile compounds were investigated in this study to observe the quality of the essential oil. It was discovered that a greater amount of overfilled solvent resulted in thicker essential oil and a longer storage time resulted in a lower amount of total oil yield. It could be seen that almost all extraction conditions did not have any significant difference in antioxidant and antimicrobial potential. The essential oil contained primary compounds such as indole, 9-tricosene, α-farnesene, muurolene, and benzyl alcohol. This study led to the conclusion that the amount of overfilled solvent from SFE affected the thickness of jasmine essential oil and its tentative volatile compounds. The longer storage time caused the significantly lower essential oil yield, but changing the extraction conditions had no significant effect on antioxidant or antimicrobial potential.

## 1. Introduction

Recently, more people have taken an interest in the recognition of aromatherapy as a nature-friendly therapy for anti-anxiety, anti-cancer, anti-diabetic and anti-inflammatory purposes [1,2]. Essential oils from some different plants were studied for antifungal activities [3], and essential oils from flowers are being acknowledged all across the world as antimicrobials for food preservation agents [4]. All of these studies have demonstrated the utility of using essential oils in our daily lives. Additionally, a lot of studies have revealed that some flower essential oils have antioxidant and antimicrobial potential, such as the *Michelia champaka* flower [5], the *Citrus aurantium* L. flower [6], and the *Jacaranda* flower [7]. They all agree that essential oil extracts from these flowers have the potential for antioxidant and antimicrobial properties for medical and pharmaceutical applications. It was found that various edible flowers contain a great variety of natural bioactive compounds [8]. However, there was also another study concluding that jasmine absolute essential oil had excellent antioxidant properties among the other 25 commercially available essential oils [9].

Jasmine is one of the world’s most widely cultivated plants due to its attractive and sweetly fragrant flowers, which have been the subject of extensive research in the pharmaceutical and therapeutic industries [10,11]. Jasmine oil extract also showed the potential for anti-inflammatory activity [12], anticancer activity toward skin and brain cancer cell lines [13], antibacterial activity [14], antiviral activity [15] and antioxidant activity [16]. Additionally, it is a choice for the perfumery industry due to its fine, sweet and elegant fragrance [17] and it can be utilized in skin care products as a scent in the cosmetics industry [18]. However, extracting essential oil from jasmine flowers is not an easy task. The extraction of essential oils from flowers remains a challenge that needs to be addressed.

Different extraction techniques, such as liquid–liquid extraction, solid-phase extraction and supercritical fluid extraction, have been employed to obtain extracts for analysis and potentially for medical applications [19,20]. Steam distillation, ethanol extract and supercritical carbon dioxide could be used for flower extraction [21,22,23], but solvent residue, supplied heat for extraction processes and extreme pressure seem to have resulted in impurities and a distorted odor of essential oil extracts. However, there is still an interesting extraction method called subcritical fluid extraction that uses a solvent that evaporates below room temperature at atmospheric pressure, such as refrigerants or butane. The raw material and the essential oil will not reach a high temperature that might damage volatile compounds in the essential oil during the extraction process.

Since the last decade, 1,1,1,2-tetrafluoroethane (HFC-134a or R134a) has been introduced as a solvent for the extraction of valuable natural compounds from aromatic and medicinal plants. HFC-134a is close to an ideal solvent for extraction because it becomes liquid when it is compressed at moderate pressure and changes to gas at atmospheric pressure. The liquid phase of HFC-134a can be used as a solvent and can be separated from the extracts by changing to the gas phase at room temperature. The advantages and limitations of using HFC-134a as a solvent compared to other methods have been discussed [24]. The potential of using HFC-134a as a solvent was summarized in the study of rose extracts with various chemical compounds [25] that could be applied to jasmine oil extraction.

Parameters of the extraction process, such as time, temperature and types of solvent, are necessary for process optimization. It was shown in the previous study that the longer the extraction time, the more yield was extracted from the raw materials [26]. However, at a certain point, extraction time did not have much effect on yield, while long storage time significantly reduced the scent intensity of flowers [27]. A response surface from varying supercritical carbon dioxide extraction temperature and pressure has an effect on extraction yield [28,29]. Additionally, different solvents also have a significant effect on the yield of extracts from dried lavender [30].

More extraction parameters of jasmine flowers have to be investigated to maximize essential oil yield based on all of the previous studies discussed above on jasmine and the extraction process and optimization of extraction parameters. Not only the quantity but also the quality of the jasmine essential oil has to be measured. The subcritical HFC-134a extraction of *Jasminum sambac* (L.) Ait. (Family: Oleaceae) was investigated at varying amounts of overfilled solvent and storage time, which have never been carefully observed to the best of our knowledge. This study hypothesized that the larger amount of overfilled solvent could extract more essential oil due to more solvent passing through the flowers, and the longer storage time could lower the amount of essential oil yield due to the essential oil in the flowers evaporating during the storage. Antioxidant activities, antimicrobial activities and tentative volatile compounds by GC-MS were conducted to measure the quality of essential oil extracts, because some differences in the quality of the essential oil were expected from the varying methods of extraction. Finally, an appropriate extraction method of subcritical HFC-134a for jasmine flowers will be provided in this study.

## 2. Results

### 2.1. Essential Oil Yield

**Figure 1:**
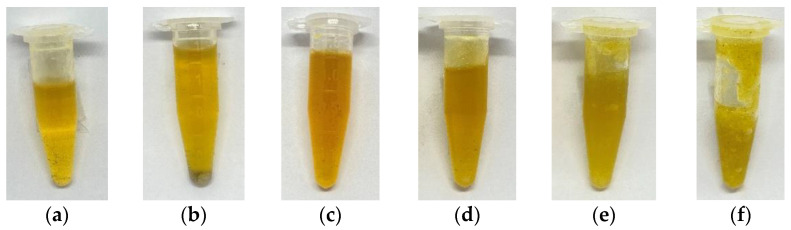
Essential oil at varying percentages of overfilled solvent: (**a**) 0%; (**b**) 10%; (**c**) 20%; (**d**) 30%; (**e**) 40%; (**f**) 50%.

In the study, essential oil was collected from a collector vessel after the extraction of *Jasminum sambac*. The amount of overfilled solvent was varied from 0% to 50% to observe the texture and yield of the essential oil. For the essential oil texture, it was found that varying the percentage of overfilled solvent could have some effect on the texture of the essential oil, as demonstrated in Figure 1. The condition of 0% overfilled solvent indicated that there was no additional solvent supplied to the extraction chamber during the extraction process. The essential oil from the flowers was light and yellowish. The condition of 50% overfilled solvent referred to 50% of the solvent being supplied to the chamber during the extraction process, and the overfilled solvent was evaporated and recovered by a recovery pump. The essential oil from the 50% overfilled solvent condition was thick and greenish-yellow. The higher the percentage of overfilled solvent, the thicker the essential oil. It could be concluded from the result that the overfilled solvent made the essential oil thicker.

**Figure 2:**
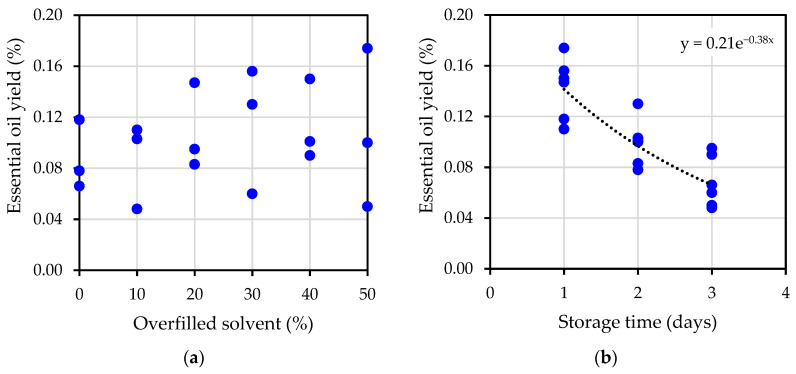
Essential oil yield: (**a**) at varying percentage of overfilled solvent; (**b**) at varying storage time.

The results of essential oil yield at varying percentages of overfilled solvent are demonstrated in Figure 2a. The yield seems to be scattered around 0.1%. It can be concluded from the results that the amount of overfilled solvent did not affect the yield. However, the more the raw material tank is overfilled during the extraction process, the more power the recovery pump needs to recover the evaporated solvent and return it to the tank. The same data set of essential oil yield was plotted with varying storage time in Figure 2b, but without considering the percentage of overfilled solvent. The graph shows a monotonic trend in essential oil yield. The longer jasmine flowers were refrigerated, the less essential oil was extracted.

### 2.2. Antioxidant Activities

#### 2.2.1. Measurement of Total Phenolic Content

**Figure 3:**
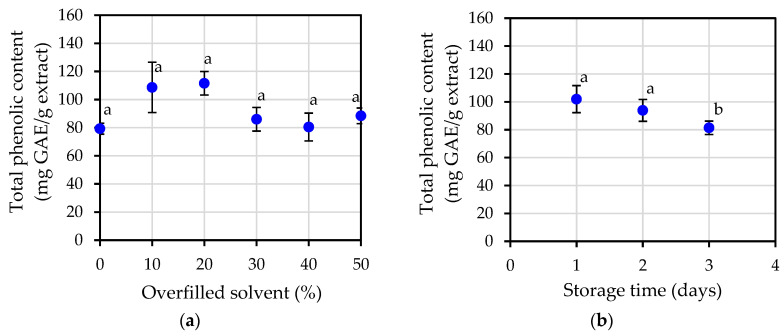
Total phenolic content of jasmine essential oil: (**a**) at varying percentage of overfilled solvent; (**b**) at varying storage time. Different letters a, b in the same figure indicate that values are significantly different (*p* < 0.05).

The total phenolic content was employed as an indicator to determine the potential of the essential oil because it was observed that essential oil extracted from jasmine flowers possessed antioxidant potential. The findings presented in Figure 3a demonstrated that different percentages of overfilled solvent show a similar amount of total phenolic content at an average value of 92.41 ± 4.66 mg GAE/g extract, with the exception of 10% overfilled, for which the values seem to be slightly lower than those found in other conditions. In addition, the various storage times, as shown in Figure 3b, did not reveal any differences in the total phenolic content at any of the measured values.

#### 2.2.2. Measurement of Total Flavonoid Content

**Figure 4:**
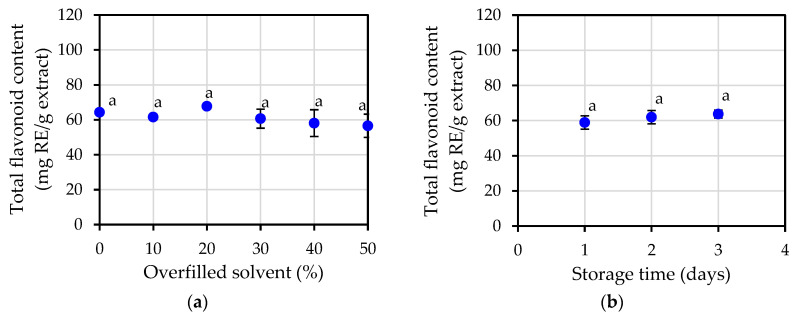
Total flavonoid content of jasmine essential oil: (**a**) at varying percentage of overfilled solvent; (**b**) at varying storage time. The same letters a in the same figure indicate that values are not significantly different (*p* < 0.05).

Another indicator that was employed in the process of determining the jasmine flower essential oil’s antioxidant capacity was the total flavonoid content. The total flavonoid content, as shown in Figure 4a,b in each of the experimental circumstances, was nearly constant with an average value of 61.50 ± 1.88 mg RE/g extract being recorded. It demonstrates that the value of the essential oil’s total flavonoid content was unaffected by either the amount of overfilled solvent or the storage time.

#### 2.2.3. Antioxidant Activity by FRAP Assay

**Figure 5:**
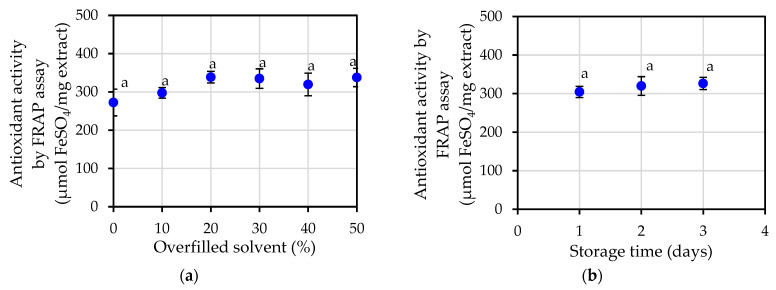
Antioxidant activity by FRAP assays of jasmine essential oil: (**a**) at varying percentage of overfilled solvent; (**b**) at varying storage time. The same letters a in the same figure indicate that values are not significantly different (*p* < 0.05).

The results of the FRAP assay on the antioxidant activity of jasmine essential oil, which can be seen in Figure 5a,b, did not have an effect on the differences in the antioxidant potential that resulted from the various extraction conditions and the amount of storage time. The FRAP assay showed an average value of 316.79 ± 10.41 µmol FeSO_4_/mg of extract for the measurement of antioxidant activity.

#### 2.2.4. Antioxidant Activity by DPPH Assay

**Figure 6:**
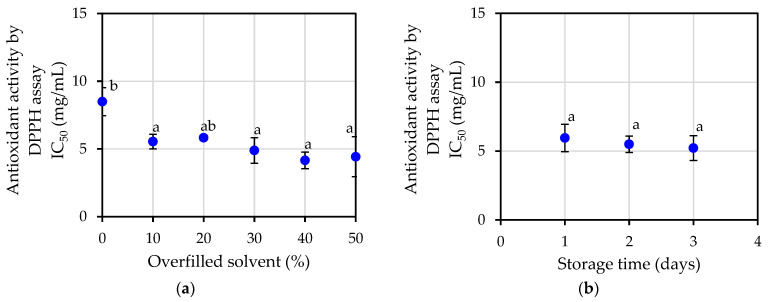
Antioxidant activity by DPPH assays of jasmine essential oil: (**a**) at varying percentage of overfilled solvent; (**b**) at varying storage time. Different letters a, b in the same figure indicate that values are significantly different (*p* < 0.05).

The value of antioxidant activity determined by the DPPH assay also demonstrated that the antioxidant potential obtained from each extraction condition behaved in a similar way. Figure 6a shows the result, which reveal that the average IC_50_ value was 5.55 ± 0.46 mg/mL. The majority of the extraction conditions revealed similar values, with the exception of 0 percent of the solvent being overfilled. Additionally, the results of the storage time as shown in Figure 6b did not appear to have any effect on IC_50_ value.

#### 2.2.5. Antioxidant Activity by ABTS assay

**Figure 7:**
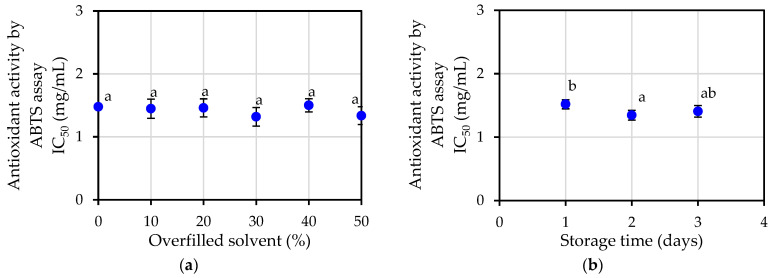
Antioxidant activity by ABTS assay of jasmine essential oil: (**a**) at varying percentage of overfilled solvent; (**b**) at varying storage time. Different letters a, b in the same figure indicate that values are significantly different (*p* < 0.05).

Similar to other antioxidant indicators, the antioxidant activity evaluation by ABTS assay showed the comparable pattern of other antioxidant measuring methods for both increasing the storage time, as shown in Figure 7a, and varying the percentage of overfilled solvent, as shown in Figure 7b. The IC_50_ value of antioxidant activity from ABTS assay was found to be 1.42 ± 0.05 mg/mL on average.

### 2.3. Antimicrobial Activity

Minimum Inhibitory Concentration (MIC) testing was used to examine the antimicrobial activity of jasmine essential oil in order to determine its potential to inhibit the growth of microorganisms as shown in Table 1. In this study, two distinct extraction conditions, 10% overfilled solvent and 50% overfilled solvent, which represented the light essential oil and the thick essential oil, were chosen. The obtained results showed that jasmine essential oil successfully inhibited the growth of all the tested microbial strains. The concentration range for the minimum inhibitory concentration started at 1.56 mg/mL for *B. subtilis* in both 10% and 50% overfilled solvent, and went up to 12.50 mg/mL for *S. aureus* in 10% overfilled solvent and 12.50 mg/mL for *S. aureus* and *E. coli* in 50% overfilled solvent. For the 10% overfilled solvent, the highest MIC values were obtained against the Gram-positive bacteria *S. aureus*, followed by *L. innocua* and *M. luteus* with the same value for the Gram-negative bacteria *E. coli*, followed by *B. subtilis*. The MIC values of 50% overfilled solvent were the same for all microbes, except for *E. coli* which required more jasmine essential oil than 10% overfilled solvent to inhibit.

Additionally, the testing of Minimum Bactericidal Concentration (MBC) found that all the test microbial strains required a higher concentration of jasmine essential oil than the MIC method. The MBC values of jasmine essential oil ranged from 25 to 100 mg/mL when it was tested with *L. innocua*, *M. luteus*, *S. aureus*, *B. subtilis* and *E. coli*. The MBC values of essential oil from 10% overfilled solvent were lower than the MBC values from 50% overfilled solvent for all the tested strains, except for *L. innocua*, where the MBC values were not different from the different overfilled conditions.

### 2.4. Tentative Volatile Compounds by GC-MS

Only two distinct extraction conditions (10% overfilled solvent for the light oil and 50% overfilled solvent for the thick oil) were chosen for a tentative volatile compound study by GC-MS. The results of the chemical compounds were listed in Table 2. The compounds from different overfilled solvent percentages were slightly different, but the main chemical compounds still appeared in the GC-MS profiles. At 10% overfilled condition, indole, the main compound, was found to be 20.62%, followed by α-farnesene (14.65%), cubedol (9.78%), 9-tricosene, (Z)- (8.04%), benzyl alcohol (7.82%), 3-hexen-1-ol, benzoate, (Z)- (6.14%), 9,12,15-octadecatrienoic acid, methyl ester, (Z,Z,Z)- (linolenic acid-4.73%), 1,6-octadien-3-ol, 3,7-dimethyl- (linalool-4.46%), acetic acid, phenylmethyl ester (benzyl acetate-3.38%), methyl anthranilate (2.45%), anti-phenylacetaldoxime (1.85%), tricosane (1.78%) and anti-phenylacetaldoxime (1.29%), respectively.

At 50% overfilled condition, the major compound was 25.48% benzyl alcohol instead of indole. Then, it was followed by cubedol (11.88%), indole (10.13%), α-farnesene (9.66%), 1,6-octadien-3-ol, 3,7-dimethyl- (4.82%), 9,12,15-octadecatrienoic acid, methyl ester, (Z,Z,Z)- (4.68%), 9-tricosene, (Z)- (4.48%), 3-hexen-1-ol, benzoate, (Z)- (3.21%), methyl anthranilate (3.19%), acetic acid, phenylmethyl ester (2.27%), naphthalene, 1,2,3,4,4a,5,6,8a-octahydro-7-methyl-4-methylene-1-(1-methylethyl)-, (1α,4aβ,8aα)-(σ-Cadinene-1.79%), phenylethyl alcohol (1.77%) and tricosane (1.13%), respectively.

The majority of the volatile compounds found in the essential oil were terpenes, terpenoids, hydrocarbons, esters and alcohols, which are all natural compounds without any toxic or synthetic compounds. Some of them had antioxidant potential. HFC-134a was not detected as a residue solvent in the jasmine essential oil obtained from the extraction process. After tentative volatile compounds from a 10% overfilled solvent condition were grouped and classified, terpenes and terpenoids were found to be 34.04% of the total, followed by 26.56% of phenolic compounds, 20.62% of indoles, 11.60% of hydrocarbons, 6.35% of fatty acids, and 0.24% of pyrans and esters. At 50% overfilled solvent condition, the majority of the tentative volatile compound group was phenolic compounds at 39.81%, followed by 33.62% of terpenes and terpenoids, 10.13% of indoles, 7.23% of hydrocarbons, 6.09% of fatty acids, 0.72% of pyrans, 0.10% of esters and 0.02% of alcohols, respectively.

## 3. Discussion

### 3.1. Essential Oil Yield

When the percentage of overfilled solvent was varied, the texture of the essential oil changed but the percent yield did not change. It could be observed that light essential oil might evaporate with the solvent from the collector. If the percentage of overfilled solvent is high, a lot of light essential oil will be carried over from the collector, leaving only thick essential oil in the collector. However, it seemed as if more essential oil was taken out of the flowers when a higher percentage of overfilled solvent was used. Because of this, the overall essential oil yield did not change.

When the jasmine storage time was considered, it was found that the average yield was reduced by approximately 30% per day of storage. A prediction of essential oil yield can be made from the results of storage time with exponential curve fitting. It was found that the original amount of oil yield at 0 days was 0.21%, and the decay constant was 0.38 with a unit of 1/day. This relation can be used to predict the amount of oil yield from jasmine flowers using HFC-134a subcritical extraction if the flowers have to be refrigerated for several days. It can also be concluded that essential oil should be extracted from jasmine flowers as early as possible.

The yield from subcritical HFC-134a extraction was higher than supercritical carbon dioxide extraction by approximately three times [31]. The operating pressure of supercritical carbon dioxide extraction was also enormously high, up to 300 bar, while the operating pressure of subcritical HFC-134a extraction was always below 10 bar. On the other hand, the extraction yield from hydrodistillation extraction was similar to subcritical HFC-134a extraction at approximately 0.09% [32], but the maximum temperature during the extraction process of subcritical HFC-134a extraction was only 40 °C. At a low extraction temperature, many volatile compounds seemed not to be damaged by heat.

### 3.2. Antioxidant Potential

The antioxidant potential measured by the FRAP assay and IC_50_ values from the DPPH assay and ABTS assay of jasmine essential oil from ethanol maceration extraction seemed to be stronger than subcritical HFC-134a extraction [33,34]. The values were different by approximately two orders of magnitude. However, the amount of total phenolic content was higher than in the study of commercial jasmine absolute from solvent extraction, with a reported value of 18.39 ± 0.02 mg GAE/g [9]. According to the different studies, the antioxidant potential of the essential oil could be influenced by a number of parameters, such as plant growth location, season and extraction techniques.

After five different antioxidant potential measurement methods were compared in this study (total phenolic content, total flavonoid content, FRAP assay, DPPH assay and ABTS assay), it was found that the overfilled solvent percentage and storage time did not have any significant effect on antioxidant potential. Statistical analysis indicated that almost all extraction conditions were not significantly different. When the results of antioxidant potential were considered along with the amount of essential oil yield, it could be concluded that the most appropriate extraction condition was the shortest storage time possible, such as 1-day storage to gain the highest essential oil yield.

### 3.3. Antimicrobial Activity

The potential of using jasmine essential oil as an antimicrobial substance has been studied in some previous works. Common microorganisms such as *Staphylococcus aureus* (Gram-positive) and *Escherichia coli* (Gram-negative) were investigated using the MIC method. MIC values from different studies and different extraction methods were reported differently [34,35,36,37]. However, it can be concluded that jasmine essential oil from HFC-134a extraction also has antimicrobial potential, as indicated by other previous studies.

One of the main components of jasmine essential oil, linalool, has been discussed as an antibacterial agent [38]. Linalool is a monoterpenoid alcohol that has antimicrobial activity against a variety of bacteria. Its mechanism of action involves membrane expansion, increased membrane fluidity and permeability, the disruption of membrane-embedded proteins, the suppression of respiration, and the modification of ion transport processes in bacteria.

### 3.4. Tentative Volatile Compound

The results of volatile compounds from GC-MS showed that common components such as indole, benzyl acetate and benzyl alcohol could be found in jasmine essential oil, similarly to previous studies [9,37]. In addition to hydrodistillation, a traditional extraction method, linalool, muurolol [32], and α-farnesene [39] were the major volatile compounds that were combined to form the jasmine smell. Most of the major volatile compounds were observed in solvent extraction studies and appeared to differ from different raw material sources [31,40,41]. However, to the best of our knowledge, the tentative volatile compounds from the extraction of *Jasminum sambac* by subcritical HFC-134a have never been observed.

The major compound of the 10% overfilled condition of jasmine essential oil was indole; however, it turned out to be benzyl alcohol at the 50% overfilled condition. It is more likely that indole was extracted early in the extraction process. If a larger amount of solvent was used, these compounds could be evaporated with the extra-filled solvent. On the other hand, the concentration of benzyl alcohol, one of the primary compounds that is predominantly found in jasmine essential oils, increased because of the larger amount of overfilled solvent. It could be interpreted as meaning that benzyl alcohol requires more solvent to extract. Other tentative volatile compounds of jasmine essential oil, such as cubedol and linalool, did not show any change between the 10% overfilled solvent condition and the 50% overfilled solvent condition.

The study, by varying the amount of overfilled HFC-134a, demonstrated that the concentration of some volatile compounds in the essential oil could be adjusted by modifying the extraction conditions. As a result, by varying the extraction conditions, the scent of the essential oil could be controlled by changing the compound concentrations. It also showed that HFC-134a could be used to extract primary compounds from fresh *Jasminum sambac* flowers at a low extraction temperature, unlike hydrodistillation and supercritical carbon dioxide.

In an ideal scenario, the extraction machine operates in a close loop, where all of the solvent used in the extraction process is recovered by a recovery pump and compressed back into a recovery tank. However, during this lab-scale experiment, there was a small amount of solvent leakage. If this machine is scaled up for commercial use, engineering modifications will need to be made to ensure complete solvent recovery and continuous operation.

## 4. Materials and Methods

### 4.1. Raw Plant Material

The raw material for extraction in this study was *Jasminum sambac*, sourced from a flower dealer in Bangkok’s Flower Market. The fresh buds were delivered in 1.5 kg batches and kept refrigerated a few degrees above freezing point during transport. Then, they were dried out overnight at 25 °C room temperature for approximately 10 h. After the buds were left out over night, they started blooming and releasing scent in the morning of the next day, before the extraction process. If the buds are kept below freezing point, they will become bruised and their petals will turn yellow.

### 4.2. Extraction Process

**Figure 8:**
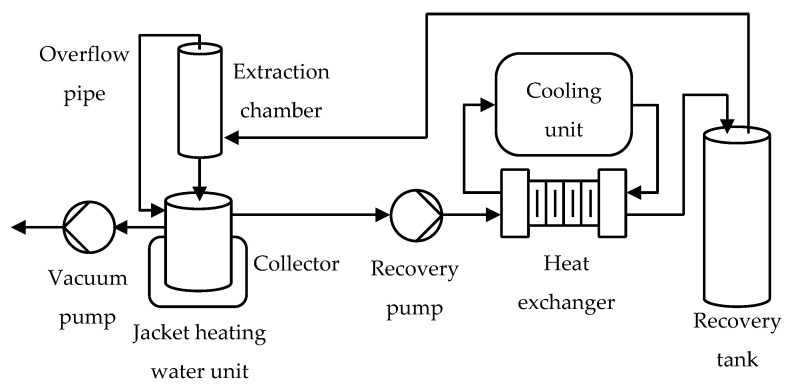
Schematic diagram of HFC-134a extraction on *Jasminum sambac*.

The HFC-134a extraction process of *Jasminum sambac* was performed in sequences and the schematic diagram is depicted in Figure 8. Firstly, blooming *Jasminum sambac* flowers as the raw material were placed in the extraction chamber, then a vacuum pump was used to remove air from the system until the pressure went down to 0.4 bar absolute. The extraction chamber was filled with the liquid phase of HFC-134a as a solvent from a recovery tank until all flowers were soaked up, as seen through an overflow pipe. Both the raw material and solvent inside the extraction chamber were held for 20 min. Additional solvent is then added to the chamber at varying amounts, up to 50% of the initial amount. Some overfilled solvent overflowed via an overflow pipe to the collector with a jacket heating water unit that automatically maintained the surrounding water temperature at 40 °C. After the solvent evaporated from the collector, a recovery pump recovered gas-phase solvent from the collector. All the gas passed through a plate heat exchanger with a cooling unit to condense the solvent back to liquid before filling back into a recovery tank. A 20-min holding period was repeated 6 times before the extraction process was completed. All of these sequences, such as the 20-min holding period and 6 overfills of solvent, were adjusted and selected until they operated under workable conditions for extraction.

### 4.3. Extraction Machine

**Figure 9:**
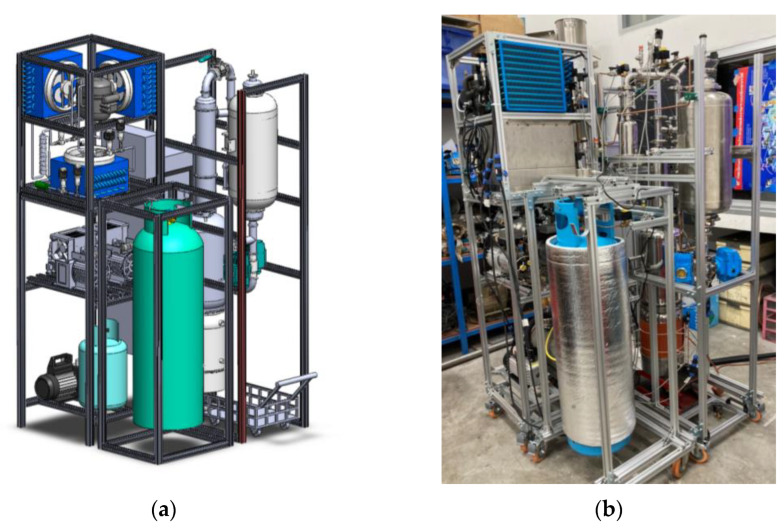
HFC-134a extractor: (**a**) CAD model; (**b**) as-built machine.

The extraction of *Jasminum sambac* was performed in an extraction machine described in Figure 9a for a CAD model and Figure 9b for an as-built machine. Mechanical components were fabricated separately from multiple suppliers and assembled in the lab. All pipes and vessels for the solvent flow were made of stainless steel and seals were made of PTFE to prevent any corrosion and contamination. The recovery pump was an oil free pump to prevent any oil contamination of the essential oil. The machine was controlled by a programmable logic controller (PLC) to fill, hold and recover solvent at specific times and in specific sequences. Temperature sensors and pressure sensors were used to indicate the status of each location and ensure the machine operated properly.

Essential oil yield was calculated based on Equation (1), which depends on the ratio of the weight of essential oil from the extraction ($m_{oil}$) and the initial weight of raw material before the extraction ($m_{raw}$).

(1) $$essential\;oil\;yield=\frac{m_{oil}}{m_{raw}}\times 100\%$$

### 4.4. Analysis

#### 4.4.1. Total Phenolic Content

The total phenolic contents of jasmine essential oil were determined using the Folin–Ciocalteu procedure [42]. In this study, 125 µL of extract solution at a concentration of 10 mg/mL was mixed with 500 µL of distilled water. The Folin–Ciocalteu reagent (125 µL) was then added. After 6 min, 1.25 mL sodium carbonate (7%) and 1 mL of distilled water were added to the mixture. The reaction mixture was incubated in the dark at room temperature for 90 min, then the absorption was measured by a 765 nm wavelength microplate reader. A calibration curve of standard reference was plotted using gallic acid (the range was from 0 to 0.5 mg/mL concentration). The results were expressed as mg gallic acid equivalent per g extract (mg GAE/extract).

#### 4.4.2. Total Flavonoid

The flavonoid content in extracts was determined spectrophotometrically, using a method based on the formation of a complex flavonoid–aluminum with a maximum absorptivity at 430 nm, as described by Djeridane et al. [43]. Rutin was used to make the calibration curve. A total of 0.1 mL of the diluted sample was mixed with 1 mL of deionized water and 1 mL of a 2% aluminum chloride methanolic solution. After incubation at room temperature for 15 min, the absorbance of the reaction mixture was measured by a spectrophotometer at 430 nm wavelength. Flavonoid content was expressed as mg rutin equivalent per gram extract (mg RE/g extract).

#### 4.4.3. FRAP Assay

The FRAP assay was measured by the method proposed [44], with some modifications. This method is based on the reduction of Fe^3+^, TPTZ (2,4,6-tripyridyl-s-triazine) complex to ferrous Fe^2+^ which forms at low pH. This reduction is followed by the measurement of the absorption change at 595 nm. All measurements were repeated three times. In the FRAP assay, a standard curve was plotted using the FeSO_4_·7H_2_O linear regression equation and used to determine the antioxidant potential from the FRAP values of the sample. The FRAP values were given in μmol FeSO_4_/mg extract.

#### 4.4.4. DPPH Assay

The 1,1-diphenyl-2-picrylhydrazy (DPPH) assay’s scavenging activity was evaluated based on previous work [45]. Briefly, 50 μL of the sample solution was mixed with 100 μL of a 0.2 mM DPPH solution dissolved in methanol. The reaction was performed in a 96-well microplate and incubated in the dark for 30 min at room temperature. The absorbance was measured at 517 nm by a microplate reader. Gallic acid and ascorbic acid were used as positive controls. The scavenging activity was expressed as the IC_50_ value (mg/mL), which is the concentration of the extract necessary to cause 50% of DPPH inhibition. The percentage of scavenging activity can be expressed as

(2) $$Scavenging\;activity\;\left(\%\right)=\frac{A_{control}-A_{sample}}{A_{control}}\times 100\%$$

when A is the measured value from the microplate reader.

#### 4.4.5. ABTS Assay

The ABTS assay was conducted following the method of Pongmalai et al. [46] and Hiranvarachat et al. [47]. ABTS+• radicals were prepared by mixing ABTS stock solution (7 mM) with 2.45 mM potassium persulfate (at a ratio of 2:1). The mixture was kept in the dark at room temperature for 16 h. The solution was diluted with ethanol until its absorbance reached 0.70 ± 0.02 at 734 nm. A total of 100 μL of the extract was mixed with 1 mL of ABTS+ solution. The absorbance was then measured at 734 nm using the spectrophotometer exactly 1 min after the mixing. Trolox was used as an antioxidant standard. The results are expressed as the IC_50_ value (mg/mL), representing the extract concentration scavenging 50% of ABTS radicals.

#### 4.4.6. Antimicrobial Activity

**Figure 10:**
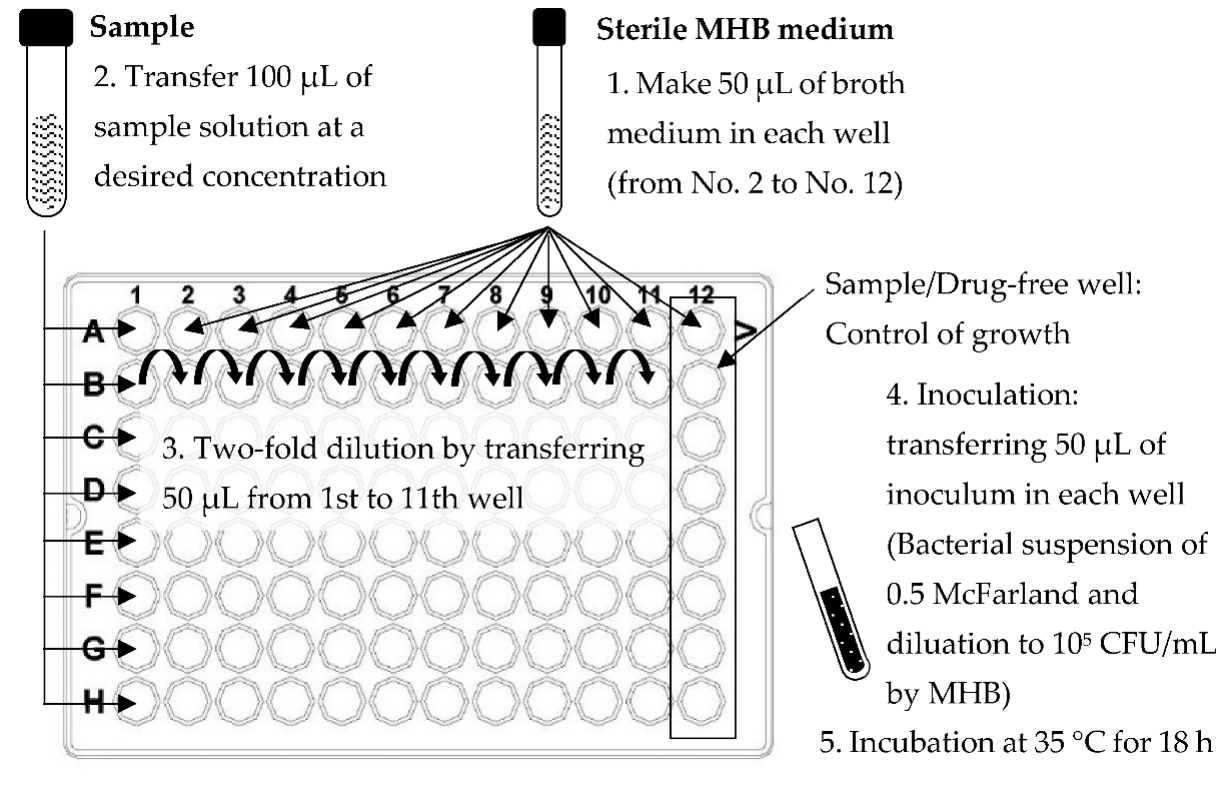
Modified broth microdilution for antibacterial testing as recommended by CLSI protocol.

The microbial testing for this study consisted of 5 bacteria species, 3 Gram-positive (*Listeria Innocua* (DMST9011 Lot.8319), *Micrococcus Luteus* and *Staphylococcus aureus* (ATCC25923 DMST8840)) and 2 Gram-negative (*Bacillus subtilis* (ATCC6633 DMST15896 Lot.8442), and *Escherichia coli* (ATCC25922 DMST4212)). The study was performed by MIC and MBC, using the microdilution method in a 96-well polystyrene plate recommended by the Clinical and Laboratory Standards Institute (CLSI) [48], with some modifications, as shown in Figure 10. After incubation at 35 °C for 18 h, 50 µL of 0.15 mg/mL resazurin was added to each well as a bacterial growth indicator. The plate was incubated at 37 °C for 1 to 4 h [49,50]. The bacterial growth was revealed by the change in color from purple to pink. The MIC value was determined as the lowest concentration that prevented a change in resazurin color [51]. The lowest concentration that inhibited the growth of bacteria was defined as the MBC.

#### 4.4.7. Tentative Volatile Profiles Analysis

The jasmine essential oil extracts were evaluated in terms of their volatile profiles by gas chromatography-mass spectrometry (GC-MS) method. The chromatographic analysis was performed on a 7890B GC system and a 7000C mass spectrometer (Agilent Technologies, Palo Alto, CA, USA) equipped with an HP-5 ms capillary column (30 m × 0.25 mm × 0.25 µm film thickness, Agilent Technologies). A total of 0.5 µL of samples diluted with ethanol were injected by an autosampler with a 25:1 split ratio. Helium was used as a carrier gas at a constant flow rate of 1.0 mL/min. The oven temperature program was initially maintained at 50 °C for 3 min, before ramping at 5 °C/min up to 250 °C and finally holding for 10 min. The temperature of the injector port and the GC-MS interface were set at 250 °C. The mass spectrometer was operated in the electron ionization (EI) mode with 70 eV electron energy, 230 °C ion source temperature, 150 °C quadrupole temperature and 33–500 m/z mass scan range.

Data acquisition and peak integration were performed using Mass Hunter Qualitative Analysis Software, version B.06.01 SP1 (Agilent Technologies). Data processing was further carried out using Microsoft Excel 2020. The tentative volatile compounds of samples were identified by comparing both their mass spectra (MS) fragmentation patterns and their linear retention indices (LRI) to those contained in the NIST2011 mass spectral library and the related literature [52]. LRI value was calculated from the retention time of each peak in sample relation to retention time of n-alkanes (C7-C33) under the same conditions according to the previous literature [53]

The quantification of each identified volatile component in terms of the relative peak area (%) was obtained by dividing the area of a single peak by the total peak areas of the total ion chromatogram (TIC).

#### 4.4.8. Data Analysis

Data are reported as (means ± standard error). They were analyzed by one-way analysis of variance and post hoc Duncan’s multiple range test. Statistical significance was considered at a *p*-value of < 0.05 [54]. All statistical analyses were performed using SPSS^®^ software (version 17.0, SPSS Inc., Chicago, IL, USA).

## 5. Conclusions

The study of subcritical HFC-134a essential oil extraction from jasmine flowers has demonstrated that the amount of overfilled solvent affected the viscosity of the essential oil. If the overfill percentage is higher, the essential oil will be thicker. However, it did not have any effect on the extraction yield, which was about 0.09%. The parameter that directly affected the amount of oil yield was the varying storage time, from one day to three days. The longer the essential oil was stored, the less essential oil yield was extracted. The decay constant was found to be 0.38, with a unit of 1/day. This extraction method could be considered an alternative method for jasmine essential oil extraction.

Antioxidant activities were investigated in this study by five different methods; measurement of total phenolic content, measurement of total flavonoid, FRAP assay, DPPH assay and ABTS assay. All of the results from these five different methods seem to point to the same conclusion; that essential oil extracts from jasmine flowers have antioxidant potential under all extraction conditions. The varying overfilled solvent percentage and storage time did not have any effect on antioxidant potential.

Antimicrobial activity was investigated by testing with five different microorganisms: *Listeria innocua*, *Micrococcus luteus*, *Staphylococcus aureus*, *Bacillus subtilis* and *Escherichia coli*. It was found that the essential oil from jasmine flowers could inhibit microorganisms. Gram-positive bacteria appeared to need more essential oil to be inhibited than Gram-negative bacteria. However, there were no observable results for the different extraction conditions between the 10% overfilled solvent and the 50% overfilled solvent.

The major components of tentatively volatile compounds were investigated at 10% and 50% overfilled solvent conditions. It was found that 20.62% of indole was the major compound in the 10% overfilled solvent condition, and 25.48% of benzyl alcohol was the major compound in the 50% overfilled solvent condition. Other common compounds, such as linalool and α-farnesene, were also found. This study demonstrated that some tentative volatile compounds in jasmine essential oils could be modified by adjusting the extraction conditions.

## Figures and Tables

**Table 1:** Antimicrobial activities of jasmine essential oil from HFC-134a extraction.

Microorganisms	*L. innocua*		*M. luteus*		*S. aureus*		*B. subtilis*		*E. coli*	
Extraction Conditions	MIC	MBC	MIC	MBC	MIC	MBC	MIC	MBC	MIC	MBC
10% overfilled (light oil)	6.25	50	6.25	50	12.50	25	1.56	25	6.25	50
50% overfilled (thick oil)	6.25	50	6.25	100	12.50	50	1.56	50	12.50	100

MIC = Minimum Inhibitory Concentration (mg/mL); MBC = Minimum Bactericidal Concentration (mg/mL); the unit of all values is [mg extract/mL DMSO].

**Table 2:** Tentative volatile compounds of jasmine essential oil at 10% overfilled solvent and 50% overfilled solvent.

RT (min)	Tentative Compound	CAS No.	LRI		Content (%)	
			Exp ^a^	Ref ^b^	10% Overfilled Solvent	50% Overfilled Solvent
3.508	Butanoic acid, 2-methyl-, methyl ester	868-57-5	-	-	-	0.02
5.200	3-Hexen-1-ol, (Z)-	928-96-1	855	-	-	0.02
9.505	3-Hexen-1-ol, acetate, (Z)-	3681-71-8	1008	1007	0.20	0.05
9.921	2H-Pyran-2-methanol, tetrahydro-	100-72-1	1022	NR	0.24	0.72
10.302	Benzyl alcohol	100-51-6	1035	1034	7.82	25.48
11.975	2-Furanmethanol, 5-ethenyltetrahydro-α, α,5-trimethyl-, cis-	5989-33-3	1089	1088	0.06	0.07
12.153	Benzoic acid, methyl ester	93-58-3	1095	1093	0.18	0.03
12.315	1,6-Octadien-3-ol, 3,7-dimethyl-	78-70-6	1100	1100	4.46	4.82
12.727	Phenylethyl Alcohol	60-12-8	1114	1116	0.48	1.77
13.460	Benzyl nitrile	140-29-4	1139	1140	0.95	0.89
14.230	Acetic acid, phenylmethyl ester	140-11-4	1165	1165	3.38	2.27
14.385	1-Ethylpentyl acetate	5921-83-5	1170	NR	0.49	0.34
15.103	Methyl salicylate	119-36-8	1195	1193	0.09	0.12
16.749	Syn-phenylacetaldoxime	7028-48-0	1253	1253	1.29	0.64
16.881	Acetic acid, 2-phenylethyl ester	103-45-7	1258	1257	0.31	0.15
17.335	Anti-phenylacetaldoxime	7028-48-0	1274	1278	1.85	0.82
17.905	Indole	120-72-9	1294	1290	20.62	10.13
19.166	Methyl anthranilate	134-20-3	1342	1341	2.45	3.19
20.024	Butyl benzoate	136-60-7	1375	1377	0.02	-
20.281	2-Propenoic acid, 3-phenyl-, methyl ester	103-26-4	1384	1388	0.03	-
20.537	Cyclohexane, 1-ethenyl-1-methyl-2,4-bis(1-methylethenyl)-, [1S-(1α,2β,4β)]-	515-13-9	1394	1394	0.11	0.11
21.255	Caryophyllene	87-44-5	1422	1420	0.04	0.04
22.109	Humulene	6753-98-6	1457	1454	0.05	0.05
22.679	Naphthalene, 1,2,4a,5,6,8a-hexahydro-4,7-dimethyl-1-(1-methylethyl)-	483-75-0	1480	1474	0.02	0.05
22.796	γ-Muurolene	30021-74-0	1484	1483	0.59	0.66
23.177	Cyclohexane, 1-ethenyl-1-methyl-2-(1-methylethenyl)-4-(1-methylethylidene)-	3242-08-8	1500	1492	0.30	0.32
23.249	α-Muurolene	10208-80-7	1503	1500	0.11	0.28
23.427	α-Farnesene	502-61-4	1510	1510	14.65	9.66
23.593	Naphthalene, 1,2,3,4,4a,5,6,8a-octahydro-7-methyl-4-methylene-1-(1-methylethyl)-, (1 α, 4aβ, 8aα)-	39029-41-9	1517	1513	0.31	0.65
23.812	Naphthalene, 1,2,3,5,6,8a-hexahydro-4,7-dimethyl-1-(1-methylethyl)-, (1S-cis)-	483-76-1	1527	1523	0.66	1.79
24.027	Naphthalene, 1,2,3,4,4a,7-hexahydro-1,6-dimethyl-4-(1-methylethyl)-	16728-99-7	1536	1536	0.04	0.06
24.152	Naphthalene, 1,2,4a,5,6,8a-hexahydro-4,7-dimethyl-1-(1-methylethyl)-,[1R-(1α,4aα,8aα)]-	17627-24-6	1541	1541	0.09	0.15
24.737	Cycloheptane, 4-methylene-1-methyl-2-(2-methyl-1-propen-1-yl)-1-vinyl-	1000159-38-5	1566	NR	0.97	0.45
24.911	3-Hexen-1-ol, benzoate, (Z)-	25152-85-6	1573	1573	6.14	3.21
25.070	Cubedol	23445-02-5	1580	1580	9.78	11.88
25.225	E-2-Hexenyl benzoate	76841-70-8	1587	1588	0.28	0.16
25.459	α-acorenol	28296-85-7	1596	1598	0.05	0.11
25.716	4-epi-cubedol	1000374-16-0	1608	1590	0.03	0.08
26.558	τ-Cadinol	5937-11-1	1646	1644	0.18	0.59
26.867	1-Naphthalenol, 1,2,3,4,4a,7,8,8a-octahydro-1,6-dimethyl-4-(1-methylethyl)-, [1S-(1α, 4α, 4aβ, 8aβ)]-	19435-97-3	1659	1645	0.14	0.65
27.355	Methyl jasmonate	1211-29-6	1681	1673	-	0.04
28.884	Benzoic acid, heptyl ester	93-99-2	1752	1753	0.49	0.26
29.205	Benzyl Benzoate	120-51-4	1767	1763	0.35	0.31
30.777	2,6,10-Dodecatrien-1-ol, 3,7,11-trimethyl-, acetate, (E,E)-	4128-17-0	1843	1843	0.04	0.03
31.071	Benzoic acid, 2-phenylethyl ester	94-47-3	1857	1860	0.20	0.26
31.354	Benzoic acid, 2-hydroxy-, phenylmethyl ester	118-58-1	1871	1870	0.04	0.07
32.453	Hexadecanoic acid, methyl ester	112-39-0	1927	1926	0.73	0.58
34.463	1,3,6,10-Cyclotetradecatetraene, 3,7,11-trimethyl-14-(1-methylethyl)-, [S-(E,Z,E,E)]-	1898-13-1	2032	2034	0.80	0.74
35.331	10-Henicosene	95008-11-0	2079	2060	0.25	0.12
35.630	9,12-Octadecadienoic acid (Z,Z)-, methyl ester	112-63-0	2095	2092	0.22	0.36
35.754	9,12,15-Octadecatrienoic acid, methyl ester, (Z,Z,Z)-	301-00-8	2102	2105	4.73	4.68
36.219	Methyl stearate	112-61-8	2128	2128	0.67	0.47
37.069	1-Docosene	1599-67-3	2176	2188	0.22	-
38.803	9-Tricosene, (Z)-	27519-02-4	2277	2274	8.04	4.48
39.184	Tricosane	638-67-5	2299	2300	1.78	1.13
40.457	9-Tetracosene	52078-54-3	2377	2378	0.40	0.14
40.820	Tetracosane	646-31-1	2399	2400	0.37	0.32
42.398	Pentacosane	629-99-2	2500	2500	0.54	0.84
45.854	Heptacosane	593-49-7	2699	2700	-	0.20
48.986	Squalene	111-02-4	2826	2847	0.32	0.21

NR = Not report; ^a^ Exp = Experimental linear retention indices calculated using n-alkane standards on a HP-5 column; ^b^ Ref = Linear retention indices were obtained from National Institute of Standards and Technology (NIST) database (webbook.nist.gov/chemistry).
